# Sulfoxidation of pyrimidine thioate derivatives and study their biological activities

**DOI:** 10.1038/s41598-024-83050-x

**Published:** 2025-01-06

**Authors:** Atif A. El-Gharably, A. A. Nassar, N. M. El-Ganzory, Khalil M. Saad-Allah, A. A. El-Barbary

**Affiliations:** 1 Chemistry Department, Faculty of Science, Tanta University, Tanta, 31527 Egypt; 2 Chemistry Department, Faculty of Science, Menoufia University, Shibin El-Kom, 32511 Egypt; 3 Botany Department, Faculty of Science, Tanta University, Tanta, 31527 Egypt

**Keywords:** Benzothioates, Sulfonyl methanone, Sulfonothioates, Benzene sulfonothioates, Pyrimidine, Antimicrobial, Antioxidants, Antitumor, Chemical biology, Chemistry

## Abstract

In a quest to innovate biologically active molecules, the benzoylation of 4,6-dimethylpyrimidine-2-thiol hydrochloride (**1**) with benzoyl chloride derivatives was employed to produce a series of pyrimidine benzothioate derivatives (**2–5**). Subsequent sulfoxidation of these derivatives (**2–5**) using hydrogen peroxide and glacial acetic acid yielded a diverse array of pyrimidine sulfonyl methanone derivatives (**6–9**). In parallel, the sulfoxidation of pyrimidine sulfonothioates (**10–12**) yielded sulfonyl sulfonyl pyrimidines (**13–15**), originating from the condensation of compound **1** with sulfonyl chloride derivatives. The newly synthesized compounds underwent characterization via FT-IR, NMR, mass spectrometry, and elemental analyses. Biological screenings unveiled interesting properties: compounds **1** and **6** exhibited significant antimicrobial potency against *S. epidermidis* and *S. haemolyticus*, whereas compound **11** showed distinct insensitivity. Excitingly, compounds **12** and **6** showcased robust antioxidant activity by efficiently scavenging DPPH^•^ radical, underscoring their potential in oxidative stress mitigation. Notably, compounds **10** and **12** displayed promising anti-tumor effects, with compound **12** demonstrating superior efficacy against the MCF-7 breast cancer cell line compared to compound **10**. The study revealed a spectrum of biological activities across the synthesized derivatives, with modifications often resulting in diminished bioactivity compared to the parent compound **1**. These findings shed light on the intricate relationship between chemical modifications and biological properties, offering valuable insights for future drug discovery endeavors.

## Introduction

Pyrimidine is the parent ring system of numerous substances that are essential to biological functions^1^. Pinner was the first to refer to the unsubstituted parent unit as pyrimidine, which marked the beginning of the scientific study of the ring system. There are several examples of the pyrimidine nucleus in nature, including uric acid, nucleic acids, alkaloids from tea, coffee chocolate, and nucleotides. Amino and hydroxyl derivatives make up the bulk of naturally occurring pyrimidines^1^. A significant and widely used derivative in pharmaceuticals, functional materials, and natural products is the pyrimidine substructure^2^. Beginning with 6-trifluoromethyl pyridinone, Nagender et al.^3^ showed how to create pyrazolopyrimidine containing 1,2,3-triazoles and examined anti-bacterial inhibition against *M. luteus, S. aureus, B. subtilis, E. coli, S. aureus, P. aeruginosa, K. planticola,* and* C. albicans*. Also, triazole derivatives have been demonstrated promising antifungal efficacy against some fungi including *A. niger* and* C. albicans*^4^. According to Fang et al.^5^, some disubstituted thiophenyl pyrimidine derivatives were prepared, which show passable antibacterial efficacy against *Enterococci* and methicillin-resistant *S. aureus*. Additionally, Ahmed et al.^6^ created two series of thienopyrimidinyl carboxamide and thienopyrimidinones, which they evaluated for their in vitro antibacterial activity against *B. subtilis, S. aureus, E. coli,* and* S. typhi*. The desired compounds were created by treating 4,6-dimethylpyrimidine-2-thiol hydrochloride (**1**) with derivatives of benzoyl chloride and/or sulfonyl chloride, followed by oxidizing the products with glacial acetic acid and hydrogen peroxide^7,8^.

Pyrimidine-containing compounds were previously recognized as a significant category of medicinal compounds that have garnered considerable attention in the academic community, as evidenced by numerous studies. The pyrimidine ring is commonly known as a privileged structure, and compounds incorporating this ring have been extensively studied for their diverse biological activities, including antimicrobial^9^, anti-tubercular^10^, anti-thyroid^11^, anti-inflammatory^12^, anti-cancer^13^, anti-viral^14^, antioxidant^15^, and many other biological activities. Moreover, pyrimidine serves as a fundamental component in the chemical structure of numerous commercially available drugs^6^. Furthermore, pyrimidines have emerged as a significant and captivating class of antibacterial medications, exerting a substantial influence on the realm of antibacterial chemotherapy, particularly in recent years. Broadly speaking, the mechanism of action of pyrimidine derivatives has been elucidated to involve the inhibition of various processes such as FtsZ (an essential protein for bacterial cell division) polymerization, DHFR (Gram-negative bacteria enzyme responsible for its existence) inhibition, modulation of GTPase activity, and interference with bacterial cell division. These multifaceted actions collectively contribute to the bactericidal effects of pyrimidines^6^.

Additionally, a considerable number of antioxidants featuring pyrimidine nuclei, such as riboflavin, thiamine, pyrazolopyrimidine, and triazolopyrimidines, have been synthesized to fulfill this objective^15^. Despite the existence of numerous antioxidants with a pyrimidine nucleus, only a limited selection is employed in clinical settings due to their associated toxic effects. Several pyrimidine derivatives have demonstrated promising antioxidant properties owing to the presence of electron-donating substituents on the pyrimidine nucleus^16^, which augment their ability to scavenge radicals and enhance their potency^17^. In essence, a positive correlation can be established between heightened antioxidant activity and reduced electron density within the ring system, thereby underscoring the favorable antioxidant potential of substituted pyrimidines.

Pyrimidine derivatives have demonstrated significant anti-tumor activity through various mechanisms, making them promising candidates for the development of anticancer drugs^18^. These compounds exert their effects by interfering with crucial cellular processes that are essential for tumor growth and progression. One of the primary modes of action of pyrimidine derivatives is their ability to inhibit the activity of thymidylate synthase (TS), an enzyme involved in DNA synthesis. By blocking TS, these derivatives disrupt the production of thymidine, a key nucleotide required for DNA replication. This disruption leads to DNA damage and ultimately hinders cell division, inhibiting tumor growth^19^. Additionally, pyrimidine derivatives can act as inhibitors of specific tyrosine kinases, such as epidermal growth factor receptor (EGFR) and vascular endothelial growth factor receptor (VEGFR)^20,21^. These kinases play crucial roles in promoting tumor cell proliferation, angiogenesis, and metastasis. By inhibiting these signaling pathways, pyrimidine derivatives impede the growth and survival of cancer cells, as well as inhibit the formation of new blood vessels that supply nutrients to tumors^22^. Furthermore, some pyrimidine derivatives exhibit pro-apoptotic activity by inducing programmed cell death in cancer cells. They can activate apoptotic pathways, leading to the elimination of malignant cells^23^. Collectively, the primary objective of this investigation is to assess the impact of sulfoxidation on pyrimidine thioate, resulting in the generation of diverse derivatives, and subsequently studying their antimicrobial, antioxidant, and antitumor potentials.

## Experimental

All melting points were performed by open capillary using electro electrothermal MEL-TEMP® apparatus (Barnstead International). FT-IR spectra were recorded on Bruker, Tensor 27 FT-IR spectrophotometer with frequency range 4000 cm^−1^ to 400 cm^−1^ (Central Lab, Tanta University, Egypt) with KBr pellets. NMR spectra (CDCl_3_) were measured on a Varian Mercury VX-300 (300 MHz) NMR spectrometer using TMS as internal standard; chemical shifts are reported as (ppm), s = singlet, d = doublet, t = triplet, m = multiplet (National Research Centre, Dokki Giza, Egypt). The mass spectra (MS) were recorded on GCMS/QP 1000 Ex mass spectrometer at 70 eV (National Research Centre, Dokki Giza, Egypt).

### Synthesis of 4,6-dimethyl pyrimidine-2-thiol hydrochloride (1)

A mixture of thiourea (31.5 g, 0.41 mol), acetylacetone (41 g, 0.41 mol), and concentrated hydrochloric acid (25 mL) was refluxed in ethanol (250 mL) for 4 h (tlc). The solvent was removed under vacuum and the obtained yellow crystals were crystallized from ethanol giving compound **1**, yield, 81%, m.p. 240 °C.

### General procedure for the synthesis of pyrimidine benzothioate derivatives (2–5)

A mixture of compound **1** (1 mmol), benzoyl chloride derivatives (1 mmol), and pyridine (20 mL) was refluxed for 10 h. The solvent was removed by a rotary evaporator, and the resulting products **(2–5**) were washed with distilled water, filtered off, and crystallized from DMF/H_2_O.

### General procedure for the synthesis of pyrimidine sulfonyl methanone derivatives (6–9)

A mixture of benzothioate derivatives (3 mmol), hydrogen peroxide (5 mL, 50%), and glacial acetic acid (20 mL) was stirred at 25 °C for 24 h. Then after, the solvent was removed using a rotary evaporator at 40 °C under reduced pressure. The resulting pyrimidine sulfonyl methanone derivatives (**6–9**) were washed with distilled water, filtered off, and crystallized from ethanol (Table 1).

**Table 1:** Physical and analytical data of compounds (**6–15**).

Cpd	m.p. (°C)	Yield (%)	M. F	Elemental analysis (%)			
					C%	H%	N%
**6**	121–123	53	C_13_H_12_N_2_O_3_S	MS; m/z Calcd: 276.31			
				Found: 275.02			
**7**	169–171	63	C_14_H_14_N_2_O_3_S	Calcd:	57.92	4.86	9.65
				Found:	58.31	4.77	9.86
**8**	238–240	82	C_13_H_11_ClN_2_O_3_S	Calcd:	50.24	3.57	9.01
				Found:	50.87	3.63	9.22
**9**	231–232	45	C_13_H_11_N_3_O_5_S	MS; m/z Calcd: 321.31			
				Found: 321.41			
**10**	Over 300	60	C_12_H_12_N_2_O_2_S_2_	Calcd:	51.41	4.31	9.99
				Found:	51.52	4.63	10.43
**11**	Over 300	68	C_13_H_14_N_2_O_2_S_2_	Calcd:	53.04	4.79	9.52
				Found:	53.65	5.05	9.74
**12**	137–139	65	C_8_H_12_N_2_O_2_S_2_	Calcd:	41.36	5.21	12.06
				Found:	42.39	5.43	12.62
**13**	Over 300	40	C_12_H_12_N_2_O_4_S_2_	MS; m/z Calcd: 312.36			
				Found: 312.44			
**14**	Over 300	45	C_13_H_14_N_2_O_4_S_2_	MS; m/z Calcd: 326.39			
				Found: 326.69			
**15**	Over 300	43	C_8_H_12_N_2_O_4_S_2_	Calcd:	36.35	4.58	10.60
				Found:	37.98	4.74	10.89

### General procedure for the synthesis of pyrimidine sulfonothioate derivatives (10–12)

A mixture of compound **1** (14 mmol), sulfonyl chloride derivatives (14 mmol), and pyridine (20 mL) was refluxed for 10 h. The solvent was removed by a rotary evaporator, and the resulting products (**10–12**) were washed with distilled water, filtered off, and crystallized from DMF/H_2_O (Table 1).

### General procedure for the synthesis of sulfonyl sulfonyl pyrimidine derivatives (13–15)

A mixture of pyrimidine sulfonothioate derivatives (**10–12**) (3.5 mmol), hydrogen peroxide (7 mL, 50%), and glacial acetic acid (20 mL) was stirred at 25 °C for 24 h. Then after, the solvent was removed using a rotary evaporator at the preceding conditions. The resulting products (**13–15**) were washed with distilled water, filtered off, and crystallized from DMF/H_2_O (Table 1).

### Biological activity of the prepared pyrimidine compounds

#### Acquisition of microbial strains in the present study

A total of eight bacterial strains and one unicellular fungal strain were acquired from the culture collection of the Botany and Microbiology Department, Faculty of Science, Tanta University, Egypt. These strains included four Gram-negative strains: *Escherichia coli* (EIEC), *Escherichia coli* (ATTC), *Klebsiella pneumoniae*, and *Pseudomonas aeruginosa*, and four Gram-positive strains: *Staphylococcus epidermidis*, *Staphylococcus haemolyticus*, methicillin-resistant *Staphylococcus aureus* (MRSA), and *Proteus vulgaris*. The single-celled yeast *Candida albicans* represented the fungal strain used in this investigation. Subsequently, the clinical strains were expeditiously transported to the Microbiology Unit, Faculty of Science, Tanta University, for conducting in vitro assessments of the antimicrobial activity of synthesized pyrimidine derivatives.

#### Assessment of antimicrobial activity for synthesized pyrimidine derivatives

The susceptibility of eight multidrug-resistant bacterial strains and one unicellular fungal strain toward 7 pyrimidine derivatives was evaluated using the well-diffusion method for determining the diameter of the inhibition zone. The microbial strains under investigation were initially sub-cultured overnight nutrient broth (for bacterial strains) or Sabouraud broth (for *Candida albicans*), followed by adjustment to achieve a turbidity equivalent to 10^6^ colony-forming units (CFU)/ml, as determined by a spectrophotometer set at 630 nm. Next, a 100 μl volume of the broth cultures was evenly spread onto nutrient agar medium (for bacterial strains) or Sabouraud agar medium (for *Candida albicans*) using a sterile swab. The plates were allowed to sit at room temperature for 10 min. to facilitate the adherence of microbial strains to the respective medium. A sterilized cork porer was used to form 8.0 mm wells in solid medium plates. The synthesized pyrimidine derivatives were prepared as solutions employing dimethyl sulfoxide (DMSO). The concentration of each pyrimidine derivative solution was set at 60 mg/ml. In aseptic conditions, 40 μl of each pyrimidine derivative solution was pipetted into the wells using a sterile automated micropipette. All plates were then incubated at 37 °C for 24 h. After the incubation period, the diameter of the inhibition zones formed around the wells was measured in millimeters (mm). These measurements were compared to the negative control (DMSO) as well as the positive antibacterial control (Ciprofloxacin) and the positive antifungal control (Itraconazole). The mean inhibition zone diameters ± standard deviation (SD) were calculated and documented in mm after repeating the experiment three times^24^.

#### Calculation of the minimum inhibitory concentration (MIC) of the synthesized pyrimidine derivatives

The determination of the minimum inhibitory concentration (MIC) was carried out using the broth microdilution method, following the Clinical Laboratory Standards Institute guidelines as adapted by^25^. Serial dilutions of the pyrimidine derivatives being tested (at concentrations of 60.0, 30.0, 15.0, and 7.5 mg/ml) were prepared in nutrient broth and transferred into a 96-well microtiter plate. Each well received 10 μl of a working inoculum suspension, prepared to match the 0.5 McFarland standards. Two controls were included in the experiment: one with medium only, serving as a sterility control, and another with medium but no pyrimidine derivatives, serving as a control to assess the viability of the inoculum. The plates were then incubated for 24 h at 37 °C. After the incubation period, 10 μl of a 0.5% aqueous solution of triphenyl tetrazolium chloride (TTC) was added to each well, followed by a further incubation of 60 min. The plates were examined to observe any color changes. Viable microorganisms interacted with the TTC solution, resulting in a color change from no color to pink. The MIC was determined as the lowest concentration of the pyrimidine derivatives that showed no color change, indicating complete inhibition of growth.

### Determination of the antioxidant radical scavenging (DPPH^*^) activity of the selected pyrimidine derivatives

The radical scavenging activity of the selected pyrimidine derivatives in DMSO was evaluated against a solution of 2,2 diphenyl-1-picrylhydrazyl (DPPH^*^) radical in methanol (37 mg/L), following the method outlined by^26^. To perform the assay, a specific volume (0.1 mL) of each sample was combined with 3.9 mL of the DPPH^*^ solution. The mixture was vigorously shaken and then allowed to stand for 30 min. in a dark environment. The reduction in DPPH^*^ absorbance caused by the samples was measured at 517 nm and utilized to determine the radical scavenging activity of the synthesized compounds.

## In vitro assessment of the cytotoxic effects of the synthesized pyrimidine derivatives against breast cancer cell line

### Cell line, media, and chemicals utilized

The MCF-7 cell line, representative of human breast cancer cells, was procured from the esteemed American Type Culture Collection (ATCC, Rockville, MD). Chemical reagents including Dimethyl sulfoxide (DMSO), MTT (3-[4,5-dimethylthiazol-2-yl]-2,5 diphenyl tetrazolium bromide), and trypan blue dye were obtained from Sigma (St. Louis, Mo., USA). Essential laboratory supplies such as Fetal Bovine serum, DMEM (Dulbecco’s modified eagle medium), RPMI-1640 (Roswell Park Memorial Institute) medium, HEPES (4-(2-hydroxyethyl)-1-piperazine ethanesulfonic acid) buffer solution, L-glutamine, gentamycin, phosphate buffer saline (PBS) and 0.25% Trypsin-EDTA were acquired from Lonza, Verviers, Belgium.

### In vitro assay

The MCF-7 cell line, a representative model of human breast cancer cells, was cultured in RPMI-1640 medium supplemented with 10% inactivated fetal calf serum and 50 µg/ml gentamycin. The cells were maintained under standard conditions of 37 ºC in a humidified atmosphere with 5% CO_2_. Subculturing of the cells was performed biweekly to triweekly to ensure proper cell propagation.

### Evaluation of cytotoxicity through viability assay

To evaluate the potential cytotoxicity, tumor cell lines were cultured in Corning® 96-well tissue culture plates at a concentration of 5 × 10^4^ cells/well and incubated for 24 h. Then, different concentrations of the two selected pyrimidine derivatives (compounds 10 and 12) were added to the plates, with each concentration replicated three times. As controls, six wells on each plate contained media or 0.5% DMSO. After another 24-h incubation period, cell viability was assessed using the MTT test. The culture media was replaced with phenol red-free RPMI 1640 medium, and each received 10 µl of a 12 mM MTT stock solution. The plates were further incubated at 37 °C with 5% CO2 for 4 h. Subsequently, 85 µl of media was removed from the wells, and 50 µl of DMSO was added and mixed thoroughly. The plates were incubated for an additional 10 min. at 37 °C. The optical density (OD) was measured at 590 nm using a microplate reader to determine the number of viable cells.

The percentage of cell viability was calculated using the formula [(ODt/ODc)] × 100%, where ODt represented the mean optical density of wells treated with the pyrimidine derivatives and ODc represented the mean optical density of untreated cells. The anti-tumor activity of the synthesized pyrimidine derivatives was compared to that of cisplatin, a standard anti-tumor agent. To generate the survival curve for each tumor cell line treated with the investigated derivatives, a dose–response curve was plotted to visualize the relationship between compound concentration and the number of surviving cells. The 50% inhibitory concentration (IC50), indicating the concentration required to induce toxic effects in 50% of intact cells, was estimated by analyzing the dose–response curve using GraphPad Prism portable software (version 8.3.0, https://thehouseofportable.com/5862/prism-portable/)^27,28^.

## Results and discussion

**Fig. 1:**
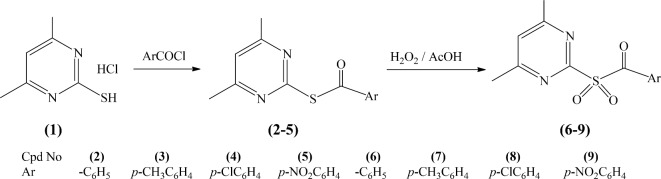
Sulfoxidation of pyrimidine benzothioate derivatives.

4,6-Dimethylpyrimidine-2-thiol hydrochloride (**1**) was synthesized by a reaction of thiourea with acetylacetone catalyzed by conc. HCl [6]. Benzoylation of compound **1** with derivatives of benzoyl chloride [6] gave pyrimidine benzothioate derivatives (**2–5**). Sulfoxidation of compounds **2–5** by hydrogen peroxide and glacial acetic acid gave derivatives of pyrimidine sulfonyl methanone (**6–9**) (Fig. 1).

### Characterization of pyrimidine sulfonyl methanone derivatives (6–9)

Sulfoxidation of (4,6-dimethylpyrimidin-2-yl)benzothioate (**2**), (4,6-dimethylpyrimidin-2-yl)-4-methyl benzothioate (**3**), (4,6-dimethylpyrimidin-2-yl)-4-chlorobenzothioate (**4**) and (4,6-dimethyl pyrimidin-2-yl)-4-nitrobenzothioate (**5**) by hydrogen peroxide and glacial acetic acid afforded (4,6-dimethylpyrimidin-2-yl)sulfonyl)(phenyl)methanone (**6**), 4,6-dimethyl pyrimidin-2-yl)sulfonyl) (p-tolyl)methanone (**7**), (4-chlorophenyl)((4,6-dimethylpyrimidin-2-yl)sulfonyl)methanone (**8**) and ((4,6-dimethylpyrimidin-2-yl)sulfonyl)(4-nitrophenyl) methanone (**9**), respectively.

**Fig. 2:**
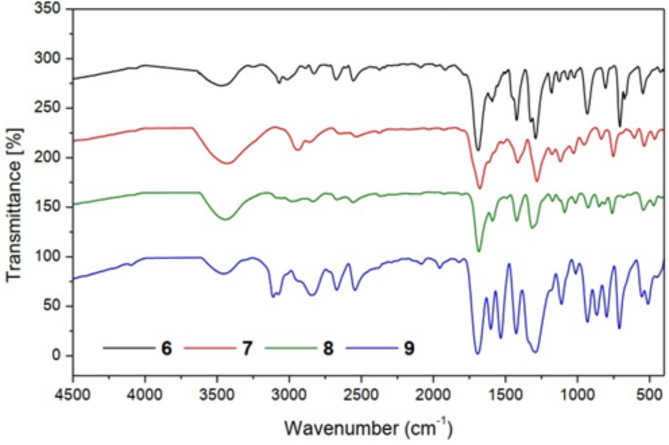
FT-IR spectra of pyrimidine sulfonyl methanone derivatives (**6–9**).

**Fig. 3:**
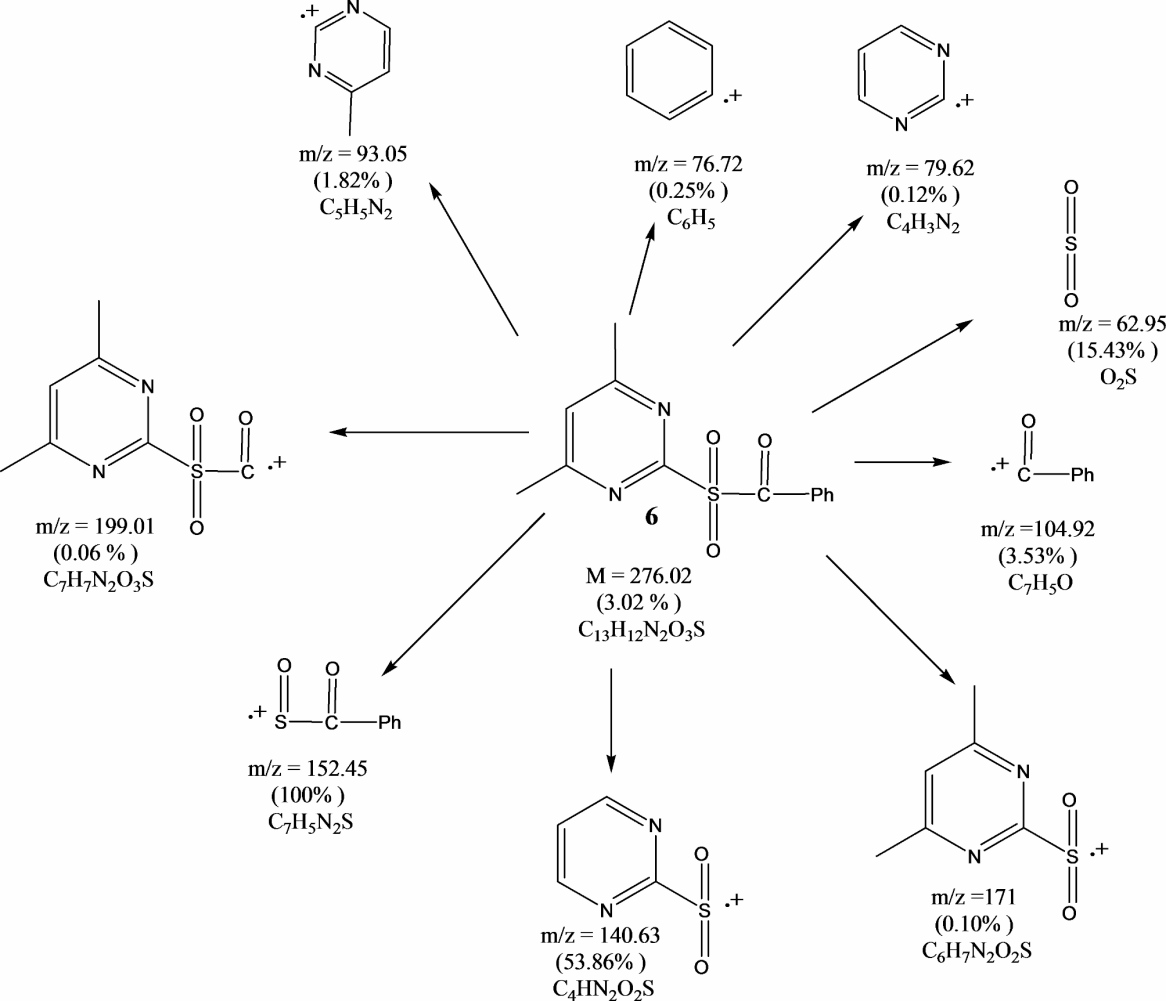
Mass fragmentations of compound **6**.

**Fig. 4:**
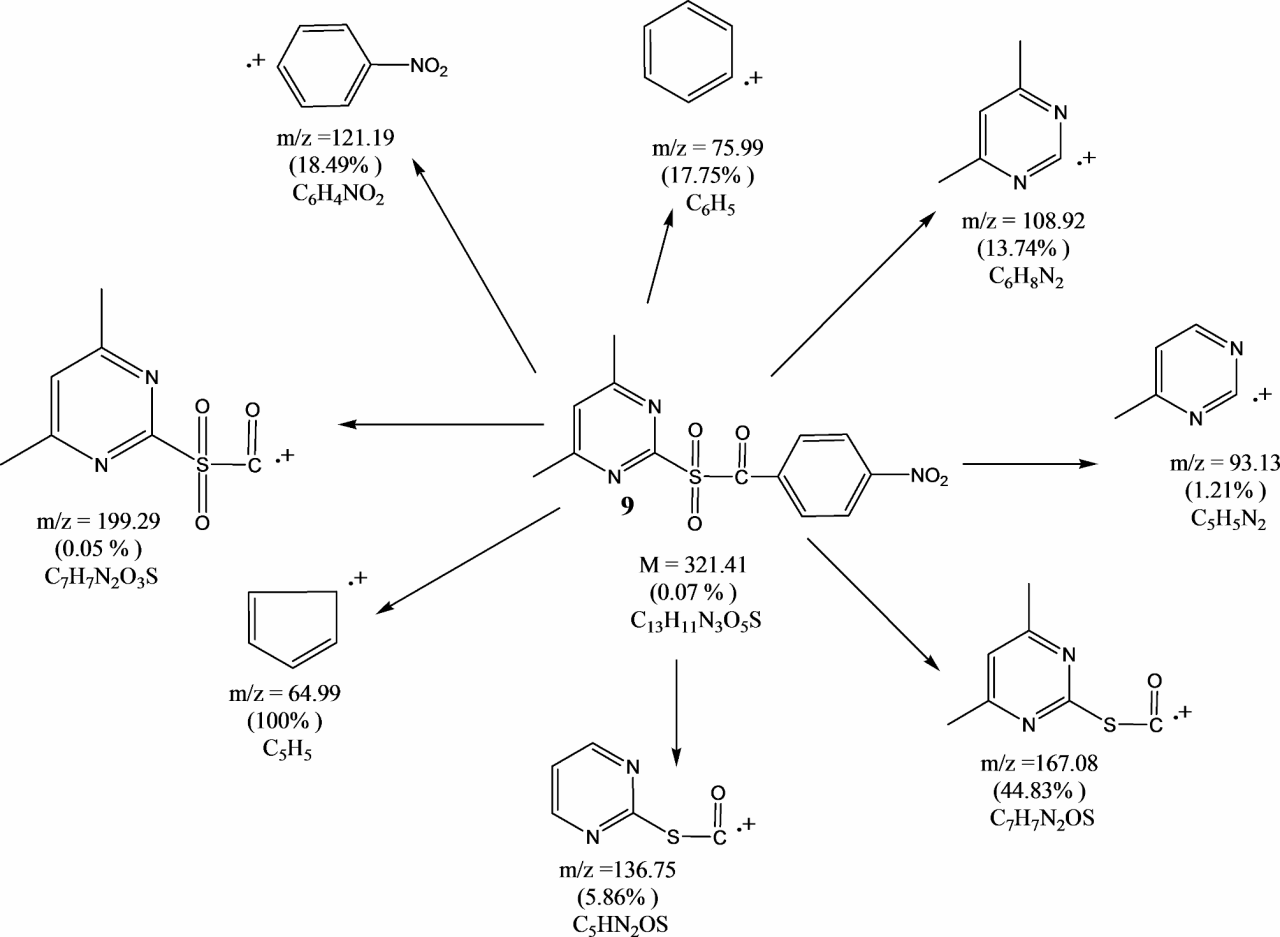
Mass fragmentations of compound **9**.

FT-IR spectra of compounds (**6–9**) showed the stretching carbonyl groups at 1690, 1679, 1685, and 1696 cm^−1^ and the sulfonyl groups at 1324, 1283, 1317 and 1292 cm^−1^, respectively (Fig. 2). ^1^H-NMR spectrum (CDCl_3_) of compound **6** illustrated the chemical shift (δ = ppm) at 2.48 (s, 6H, pyrimidine methyl protons), at 7.43 (s, 1H, aromatic protons of pyrimidine ring) and 7.45–7.91 (m, 5H, aromatic protons). ^13^C NMR (CDCl_3_) *δ*(ppm) of compound **6**: 188.22, 171.11, 163.41, 149.63, 139.82, 134.35, 131.58, 124.24 and 29.33. The mass spectrum of compound **6** (Fig. 3) showed the molecular ion peak at 276.02 (M^+^, 3.02%). Additionally, ^1^H-NMR spectrum (CDCl_3_) of compound **7** the chemical shift (δ = ppm) at 1.2 (s, 3H, tolyl methyl protons), at 2.48 (s, 6H, pyrimidine methyl protons), at 7.24 (s, 1H, aromatic protons of pyrimidine ring) and 7.27–7.82 (m, 4H, aromatic protons). ^13^C NMR (CDCl_3_) *δ*(ppm) of compound **7**: 188.88, 165.11, 158.47, 139.64, 133.82, 129.35, 126.58, 119.24, 33.39 and 19.32. Moreover, the ^1^H-NMR spectrum (CDCl_3_) of compound **8** illustrated (s, 6H, pyrimidine methyl protons) at 2.47, (s, 1H, aromatic protons of pyrimidine ring) at 7.51 and (m, 4H, aromatic protons) at 7.53–7.91 ppm. ^13^C NMR (CDCl_3_) *δ*(ppm) of compound **8**: 192.12, 174.14, 167.51, 148.65, 141.82, 137.97, 131.78, 123.99 and 29.39. ^1^H-NMR spectrum (CDCl_3_) of compound **9** illustrated (s, 6H, pyrimidine methyl protons) at 2.47, (s, 1H, aromatic protons of pyrimidine ring) at 7.41 and (m, 4H, aromatic protons) at 7.65–8.35 ppm. ^13^C NMR (CDCl_3_) *δ*(ppm) of compound **9:** 191.52, 173.25, 166.55, 147.66, 141.82, 137.75, 119.22, 114.19 and 24.34. The mass spectrum of compound **9** (Fig. 4) showed the molecular ion peak at 321.41 (M^+^, 0.07).

**Fig. 5:**
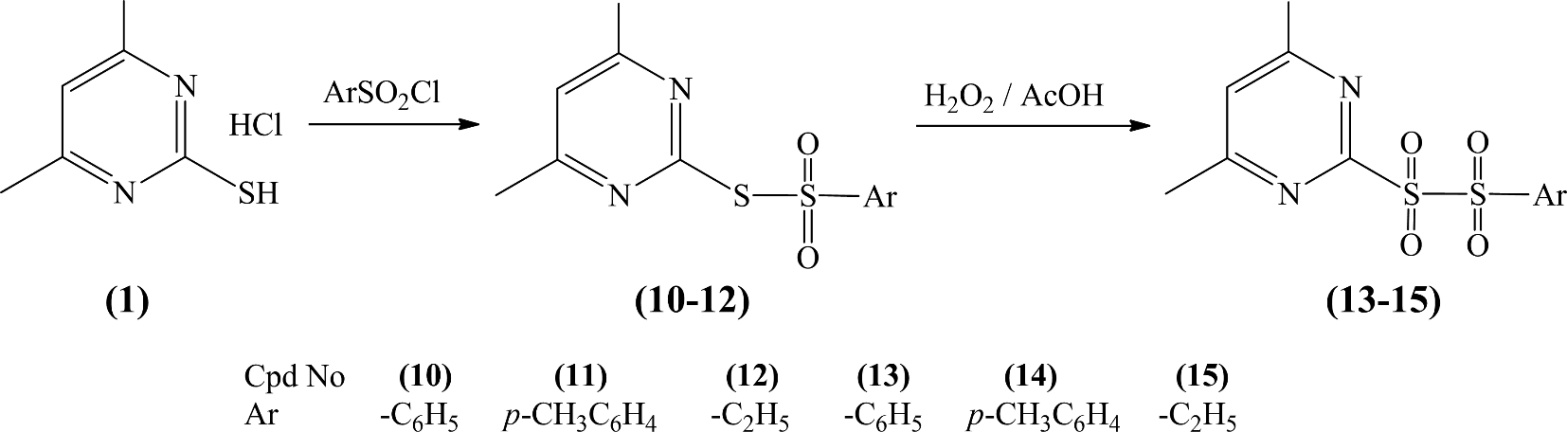
Sulfoxidation of Pyrimidine Sulfonothioate Derivatives.

Additionally, the reaction of compound **1** with sulfonyl chloride derivatives afforded pyrimidine sulfonothioate derivatives (**10–12**), which undergo sulfoxidation by hydrogen peroxide and glacial acetic acid to provide sulfonyl sulfonyl pyrimidine derivatives (**13–15**) (Fig. 5).

### Characterization of pyrimidine sulfonothioate derivatives (10–12)

Treatment of 4,6-dimethylpyrimidin-2-thiol hydrochloride (**1**) with benzene sulfonyl chloride, 4-methyl benzene sulfonyl chloride, and/or ethane sulfonyl chloride in pyridine gave (4,6-dimethylpyrimidin-2-yl)benzenesulfonothioate (**10**), (4,6-dimethylpyrimidin-2-yl)4-methyl benzenesulfono thioate (**11**) and/or (4,6-dimethylpyrimidin-2-yl)ethane sulfonothioate (**12**), respectively.

**Fig. 6:**
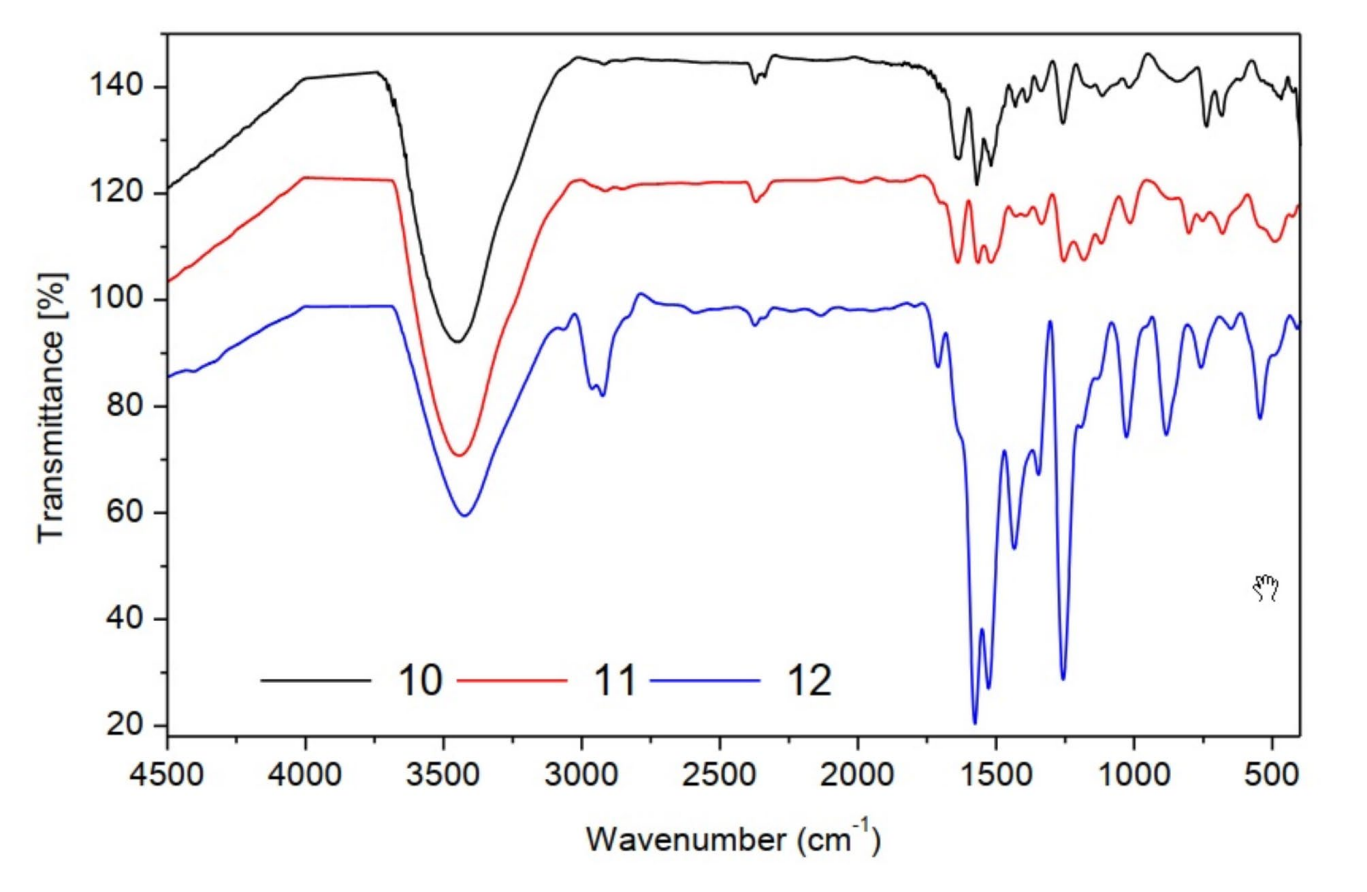
FT-IR spectra of pyrimidine sulfonothioate derivatives (**10–12**).

FT-IR spectra of compounds (**10–12**) showed sulfonyl groups at the range 1238–1325 cm^−1^ (Fig. 6). ^1^H-NMR spectrum (CDCl_3_) of compound **10** provided (s, 6H, pyrimidine methyl protons) at 2.22, (s, 1H, aromatic protons of pyrimidine ring) at 7.28 and (m, 5H, aromatic protons) at 7.35–8.82 ppm. ^1^H-NMR spectrum (CDCl_3_) of compound **11** illustrated (s, 3H, tolyl methyl protons) at 1.1, (s, 6H, pyrimidine methyl protons) at 2.44, (s, 1H, aromatic protons of pyrimidine ring) at 7.60 and (m, 4H, aromatic protons) at 7.31–7.88 ppm. ^1^H-NMR spectrum (CDCl_3_) of compound **12** showed the chemical shift (δ = ppm) at 1.00–1.18 (t, 3H, methyl protons of ethyl group), at 2.29 (s, 6H, pyrimidine methyl protons), at 4.29–4.35 (q, 2H, methylene protons of ethyl group) and 7.28 (s, 1H, aromatic protons of pyrimidine ring).

### Characterization of sulfonyl sulfonyl pyrimidine derivatives (13–15)

The reaction of pyrimidine sulfonothioate derivatives (**10–12**) with hydrogen peroxide and glacial acetic acid provided 4,6-dimethyl-2-((phenylsulfonyl)sulfonyl)pyrimidine (**13**), 4,6-dimethyl-2-(tosylsulfonyl)pyrimidine (**14**) and/or 2-((ethylsulfonyl)sulfonyl)-4,6-dimethyl pyrimidine (**15**), respectively.

**Fig. 7:**
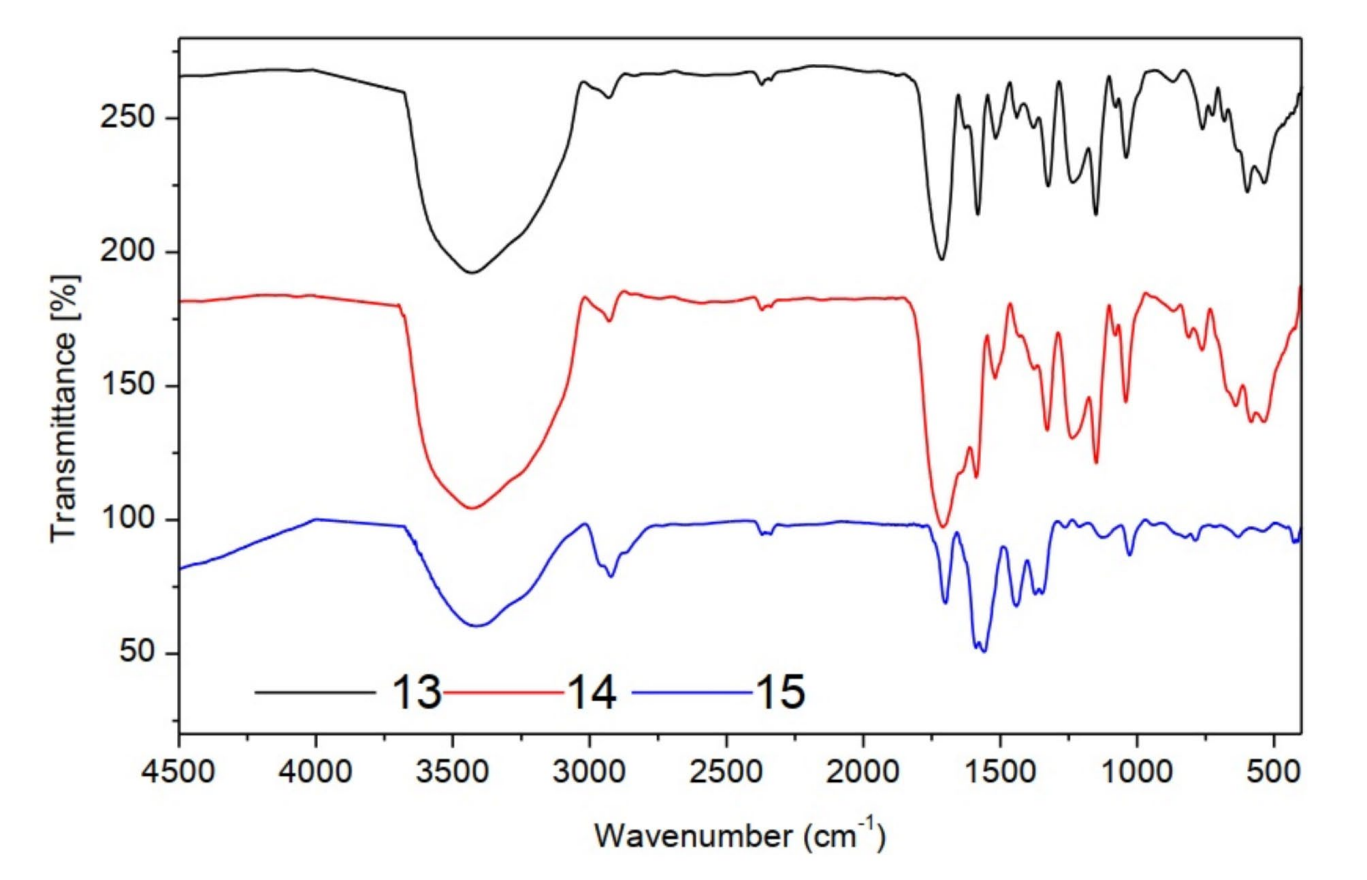
FT-IR spectra of sulfonyl sulfonyl pyrimidine derivatives (**13–15**).

**Fig. 8:**
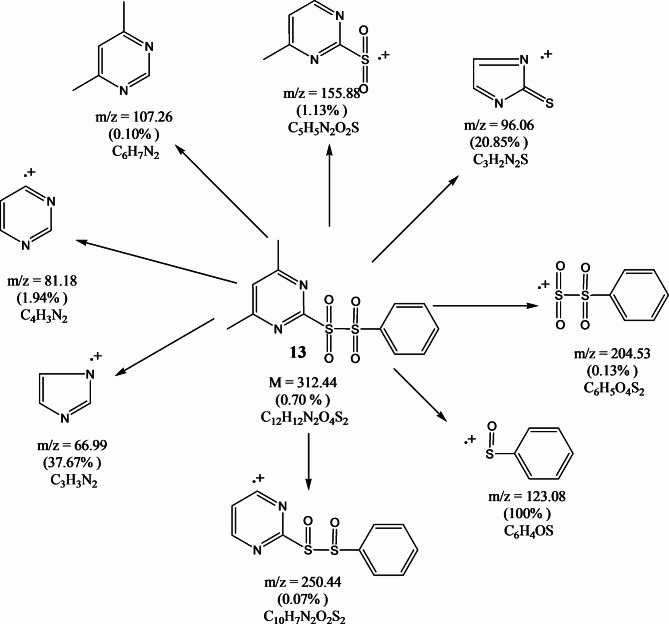
Mass fragmentations of compound **13**.

**Fig. 9:**
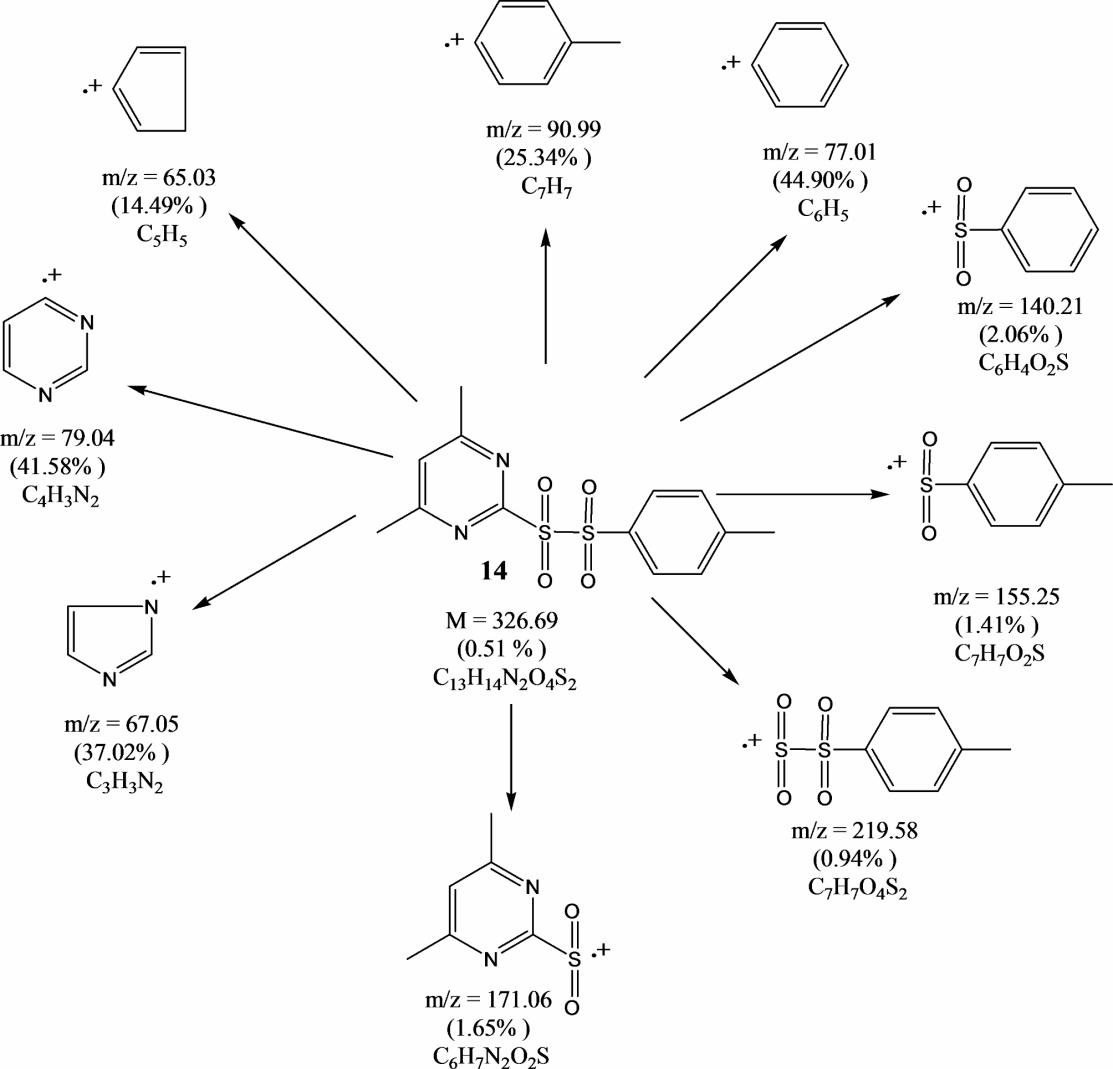
Mass fragmentations of compound **14**.

FT-IR spectra of compounds (**13–15**) showed sulfonyl groups in the range 1238–1325 cm^−1^ (Fig. 7). ^1^H-NMR spectrum (CDCl_3_) of compound **13** illustrated (s, 6H, pyrimidine methyl protons) at 2.50, (s, 1H, aromatic protons of pyrimidine ring) at 7.54 and (m, 5H, aromatic protons) at 7.47–7.92 ppm. ^13^C NMR (CDCl_3_) *δ*(ppm) of compound **13:** 167.60, 160.46, 139.82, 132.82, 129.47, 127.39, 119.89 and 40.34. The mass spectrum of compound **13 **(Fig. 8) showed the molecular ion peak at 312.44 (M^+^, 0.70%). ^1^H-NMR spectrum (CDCl_3_) of compound **14** provided (s, 3H, tolyl methyl protons) at 1.2, (s, 6H, pyrimidine methyl protons) at 2.47, (s, 1H, aromatic protons of pyrimidine ring) at 7.24 and (m, 4H, aromatic protons) at 7.00–8.16 ppm. ^13^C NMR (CDCl_3_) *δ*(ppm) of compound **14:** 178.65, 171.51, 151.81, 146.82, 141.37, 138.57, 130.99, 34.34 and 30.34. The mass spectrum of compound **14 **(Fig. 9) showed the molecular ion peak at 326.69 (M^+^, 0.51%). ^1^H-NMR spectrum (CDCl_3_) of compound **15** showed (t, 3H, methyl protons of ethyl group) at 1.10–1.28, (s, 6H, pyrimidine methyl protons) at 2.35, (q, 2H, methylene protons of ethyl group) at 3.89–4.05 and (s, 1H, aromatic protons of pyrimidine ring) at 7.32 ppm. ^13^C NMR (CDCl_3_) *δ*(ppm) of compound **15:** 171.58, 164.57, 130.99, 45.33.82, 30.44 and 09.99.

### The antibacterial activity of synthesized pyrimidine derivatives

**Fig. 10:**
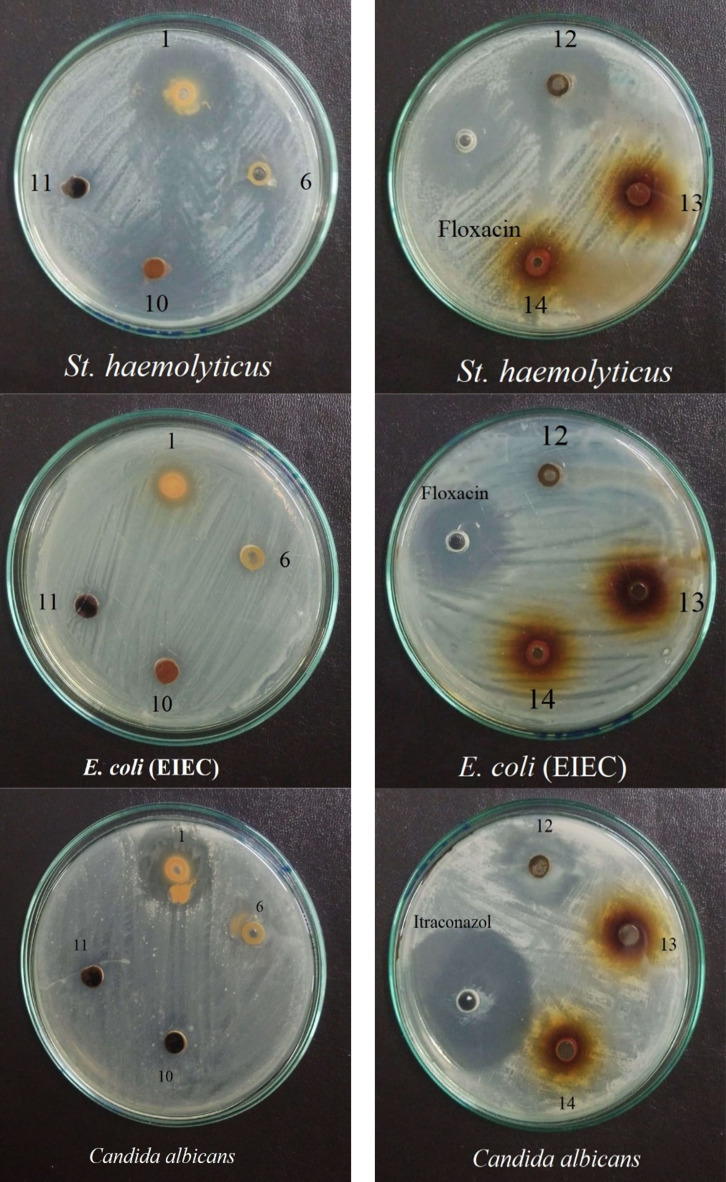
Representative images of the antimicrobial activity of the modified pyrimidine compounds.

The antimicrobial potential of the synthesized pyrimidine derivatives was evaluated against a selection of bacterial and fungal strains. The bacterial strains included human pathogenic strains *Escherichia coli* (EIEC), *Escherichia coli* (ATTC), *Klebsiella pneumoniae*, and *Pseudomonas aeruginosa* as Gram-negative bacteria. While the Gram-positive strains included *Staphylococcus epidermidis*, *Staphylococcus haemolyticus*, *Methicillin-resistant Staphylococcus aureus* (MRSA), and *Proteus vulgaris*. The fungal strain tested was *Candida albicans*. The study aimed to determine the antibacterial activity of the synthesized pyrimidine derivatives compared to the antibiotic floxacin and the antifungal activity compared to itraconazole, to assess their potential as antimicrobial agents (Table 2 and Fig. 10).

**Table 2:** Antimicrobial activity of the synthesized pyrimidine derivatives against the tested microorganisms.

Compound	*E. coli* (EIEC)	*E. coli* (ATTC)	*K. pneumoniae*	*P. aeruginosa*	*S. epidermidis*	*S. haemolyticus*	MRSA	*P. vulgaris*	*C. albicans*
1	13.67 ± 1.53^a^	14.67 ± 0.58^a^	17.33 ± 0.58^a^	15.00 ± 0.00^a^	30.33 ± 0.58^a^	31.67 ± 0.58^b^	19.67 ± 0.58^a^	14.33 ± 1.15^a^	24.67 ± 0.58^a^
6	ND^b^	11.33 ± 0.58^b^	11.33 ± 0.58^b^	11.67 ± 0.58^b^	14.33 ± 0.58^d^	17.00 ± 1.00	11.67 ± 0.58^c^	ND^b^	19.33 ± 1.15^b^
10	ND^b^	ND^c^	ND^c^	ND^c^	20.33 ± 0.58^c^	34.33 ± 1.53^a^	18.33 ± 0.58^b^	ND^b^	ND^c^
11	ND^b^	ND^c^	ND^c^	ND^c^	ND^e^	ND^d^	ND^d^	ND^b^	ND^c^
12	ND^b^	ND^c^	ND^c^	ND^c^	30.67 ± 1.15^a^	34.67 ± 0.58^a^	18.67 ± 1.15^ab^	ND^b^	25.33 ± 0.58^a^
13	ND^b^	ND^c^	ND^c^	ND^c^	20.33 ± 0.58^c^	ND^d^	ND^d^	ND^b^	ND^c^
14	ND^b^	ND^c^	ND^c^	ND^c^	22.00 ± 0.00^b^	17.33 ± 0.58^c^	ND^d^	ND^b^	ND^c^
DMSO	ND^b^	ND^c^	ND^c^	ND^c^	ND	ND	ND^d^	ND^b^	ND^c^
Flo (30 mg/ml)	29.33 ± 1.15	24.33 ± 0.58	19.33 ± 1.15	19.67 ± 0.58	41.00 ± 1.00	29.33 ± 1.15	19.33 ± 1.15	20.67 ± 1.15	–
Itc (30 mg/ml)	–	–	–	–	–	–	–	–	45.00 ± 1.00
Statistical analysis									
F	240.14	1296.66	1635.33	2725.00	861.41	1105.89	810.57	462.25	1640.22
P	0.0000	0.0000	0.0000	0.0000	0.0000	0.0000	0.0000	0.0000	0.0000
LSD at 5%	1.01	0.54	0.54	0.38	1.08	1.38	1.01	0.76	0.94

The results are represented as the mean of 3 replicates ± SD (standard deviation). Different letters within the same column represent significant differences at *P* < 5%

Out of the synthesized pyrimidine derivatives, seven compounds **1, 6, 10, 11, 12, 13,** and** 14** were specifically examined for their antimicrobial effects. Compound **1** demonstrated significant antibacterial activity against all tested microorganisms, with inhibition zone diameters ranging from 13.67 to 31.67 mm. Notably, it displayed the highest activity against *S. haemolyticus* (31.67 mm), *S. epidermidis* (30.33 mm), and *C. albicans* (24.67 mm). However, it exhibited the least activity against *E. coli* EIEC (13.67 mm), *P. vulgaris* (14.33 mm), and *E. coli* ATTC (14.67 mm). Compound **6** showed antimicrobial activity against most of the tested microorganisms, except *E. coli* EIEC and *P. vulgaris*. Its most pronounced activity was observed against *C. albicans*, *S. haemolyticus*, and *S. epidermidis*, with inhibition zone diameters of 19.33, 17.00, and 14.33 mm, respectively.

Compound **10** did not exhibit antimicrobial activity against the tested Gram-negative bacteria or the fungal strain. However, it displayed potent antibacterial activity against *S. haemolyticus, S. epidermidis,* and *MRSA*, with inhibition zone diameters of 34.33, 20.33, and 18.33 mm, respectively. Compound **11** did not show any antimicrobial activity against any of the tested microorganisms. Similarly, compounds **12, 13,** and** 14** did not demonstrate antimicrobial efficacy against the tested Gram-negative bacteria or the unicellular yeast-fungal strain *C. albicans*, except for compound **12**, which exhibited inhibition zones 25.33 mm against *C. albicans*. This compound also exhibited inhibition zones of 34.67, 30.67, and 18.67 mm against *S. haemolyticus, S. epidermidis*, and MRSA, respectively. Compound **13** only showed an antibacterial effect against *S. epidermidis* (20.33 mm), while compound **14** produced inhibition zone diameters of 22.00 and 17.33 mm against *S. epidermidis* and *S. haemolyticus*, respectively.

The antibiotic floxacin displayed relatively efficient antibacterial effects against all tested strains, with mean inhibition zone diameters ranging from 19.33 to 41.00 mm. Similarly, the antifungal agent itraconazole exhibited potent antifungal activity against *C. albicans*, with a mean inhibition zone of 45.00 mm. Additionally, the solvent used (DMSO) to prepare the compound solutions did not exhibit any antimicrobial effects against the tested microorganisms.

Pyrimidine derivatives have gained significant attention in the field of medicinal chemistry due to their diverse biological activities, including antimicrobial properties. Several studies have investigated the antimicrobial potential of pyrimidine derivatives against various microorganisms such as bacteria, fungi, and parasites^29,30^. Pyrimidine derivatives have shown promising antimicrobial effects against a wide range of bacteria, including both Gram-positive and Gram-negative strains. For instance, two studies evaluated the antimicrobial activity of some newly synthesized pyrimidine derivatives and found that they exhibited significant inhibitory effects against both methicillin-resistant *Staphylococcus aureus* (MRSA) and *Escherichia coli* strains^31,32^. Similarly, another study by Suresh et al.^33^ reported the synthesis of pyrimidine derivatives and demonstrated their potent antibacterial activity against *Bacillus cereus* and *Staphylococcus aureus* strains. Pyrimidine derivatives have also displayed notable antifungal activity against various fungal pathogens. In a study focusing on the antifungal activity of some newly synthesized pyrimidine derivatives, the evaluated compounds exhibited potent antifungal activity against *Trichophyton rubrum, Aspergillus flavus,* and *Geotrichum candidum*, indicating their potential as antifungal agents^34^. Furthermore, another study investigating the antifungal activity of pyrimidine derivatives has demonstrated their inhibitory effects against *Aspergillus flavus*, *A. fumigatus*, *Candida albicans*, and *Penicillium marneffei* strains^35^.

The antimicrobial activity of compound **1** was significantly reduced upon introducing modifications, namely the substitution by a benzoyl group (compound **6**), phenyl group (compound **10**), tolyl group (compound **11**), or through sulfoxidation (compounds **13** and **14**), as observed in the present investigation. The introduction of a benzoyl group (compound **6**) likely disrupted the molecular arrangement necessary for effective interaction with microbial targets^36^. This substitution might have altered the physicochemical properties of the compound, affecting its ability to bind to specific receptors or enzymes crucial for microbial growth and survival. Similarly, the substitution with a phenyl group (compound **10**) or tolyl group (compound **11**) could have caused steric hindrance or changes in electronic properties, leading to a compromised interaction with target molecules involved in microbial processes^37^. These modifications may have hindered the compound's ability to exert its antimicrobial effects effectively. Furthermore, the sulfoxidation process applied to compounds **13** and **14**, which involves the introduction of an oxygen atom, may have altered the overall spatial arrangement and electronic characteristics of the molecules. This change could have disrupted the structural requirements for effective binding to microbial targets, resulting in reduced antimicrobial activity.

### MIC of the most promising pyrimidine derivatives

The present study assessed the minimum inhibitory concentration (MIC) of the pyrimidine compounds to determine their effectiveness against the tested microorganisms. Various concentrations (7.5, 15, 30, and 60 mg/mL) of the compounds were prepared and tested against the pathogenic strains as outlined in (Table 3). For compound **1**, *S. epidermidis* was inhibited at a concentration of 7.5 mg/mL, while *K. pneumoniae, P. aeruginosa, S. haemolyticus* and *C. albicans* were inhibited at a concentration of 15 mg/mL. In contrast, *E. coli*, MRSA, and *P. vulgaris* required a concentration of 30 mg/mL to achieve inhibition. Compound **6** exhibited lower efficacy compared to compound **1**. It inhibited the growth of *S. epidermidis*, *S. haemolyticus*, and *C. albicans* at a concentration of 30 mg/mL, while the remaining organisms required a concentration of 60 mg/mL for inhibition.

**Table 3:** The MIC (mg/ml) values of the synthesized pyrimidine derivatives against the tested microorganisms.

Compound	*E. coli* (EIEC)	*E. coli* (ATTC)	*K. pneumoniae*	*P. aeruginosa*	*S. epidermidis*	*S. haemolyticus*	*MRSA*	*P. vulgaris*	*C. albicans*
1	30.0	30.0	15.0	15.0	7.5	15.0	30.0	30.0	15.0
6	ND	60.0	60.0	60.0	30.0	30.0	60.0	ND	30.0
10	ND	ND	ND	ND	30.0	15.0	30.0	ND	ND
11	ND	ND	ND	ND	ND	ND	ND	ND	ND
12	ND	ND	ND	ND	15.0	7.5	30.0	ND	15.0
13	ND	ND	ND	ND	30.0	ND	ND	ND	ND
14	ND	ND	ND	ND	30.0	30.0	ND	ND	ND

At a concentration of 15 mg/mL, compound **10** inhibited the growth of *S. haemolyticus*, whereas *S. epidermidis* and MRSA required a concentration of 30 mg/mL for inhibition. Compound **12** inhibited *S. haemolyticus* at a concentration of 7.5 mg/mL, while a concentration of 15 mg/mL was sufficient to inhibit the growth of *S. epidermidis* and *C. albicans*. MRSA displayed higher resistance, requiring a concentration of 30 mg/mL for inhibition by compound **12**. Similarly, compounds **13** and **14** exhibited the least efficiency in inhibiting *S. epidermidis*, as their growth was inhibited at a concentration of 30 mg/mL.

### Radical scavenging (DPPH^*^) activity of the pyrimidine derivatives

The principle behind this test is to use electron withdrawal potential from the examined compound to bleach the violet color of the DPPH^*^ radical. The more potential for donating electrons a compound has the greater radical scavenging activity it possesses. According to the findings of this study, compounds **12**, and **6** showed higher scavenging activity than the start compound **1**, as the modification made on these compounds, through ethyl and benzoyl substitution, had resulted in 127.56, and 51.10%, respectively, increases in the DPPH scavenging activity of these compounds as compared to the start compound (Table 4). Modification of the starting compound to produce compounds **13** and **14**, on the other hand, resulted in a significant reduction in their radical scavenging activity, with compound **13** having the least activity. Contrastingly, the modification made on compounds **10** and **11** resulted in complete inhibition of the radical scavenging activity. One of the most used techniques for figuring out whether a natural or synthetic chemical molecule has antiradical and antioxidant characteristics is the DPPH radical scavenging test. The utilization of the pyrimidine derivatives as antioxidants and anti-inflammatory agents is just one of their many different applications.

**Table 4:** DPPH radical scavenging activity (%) of the synthesized pyrimidine derivatives.

Compounds	DPPH activity (%)
1	27.93 ± 2.38^c^
6	42.20 ± 1.98^b^
10	0.00f.
11	0.00f.
12	63.56 ± 0.81^a^
13	19.06 ± 0.67^e^
14	23.18 ± 0.28^d^
Statistics	
F	1001.73
P	0.0000
LSD at 5%	2.171

The results are represented as the mean of 3 replicates ± SD (standard deviation). Different letters within the same column represent significant differences at *P* < 5%

Pyrimidine derivatives have demonstrated significant antioxidant activity, making them valuable compounds in the field of oxidative stress-related diseases. These derivatives possess the ability to scavenge reactive oxygen species (ROS) and inhibit oxidative damage to cellular components. The antioxidant activity of pyrimidine derivatives is primarily attributed to their structural features, such as the presence of electron-donating functional groups^38^. These groups facilitate the donation of electrons or hydrogen atoms to unstable ROS, thereby neutralizing their harmful effects. The derivatives' antioxidant properties help maintain cellular redox balance and protect biomolecules, including lipids, proteins, and DNA, from oxidative damage. Furthermore, pyrimidine derivatives have shown the ability to chelate transition metal ions, such as iron and copper, which can contribute to the generation of harmful free radicals^39^. By chelating these ions, the derivatives prevent their participation in Fenton and Haber-Weiss reactions, which generate highly reactive hydroxyl radicals. These properties make them promising candidates for the development of therapeutic agents to combat oxidative stress-related diseases and conditions. Further research is needed to explore their full potential and optimize their efficacy as antioxidants.

### The antitumor activity of some synthesized pyrimidine derivatives

**Fig. 11:**
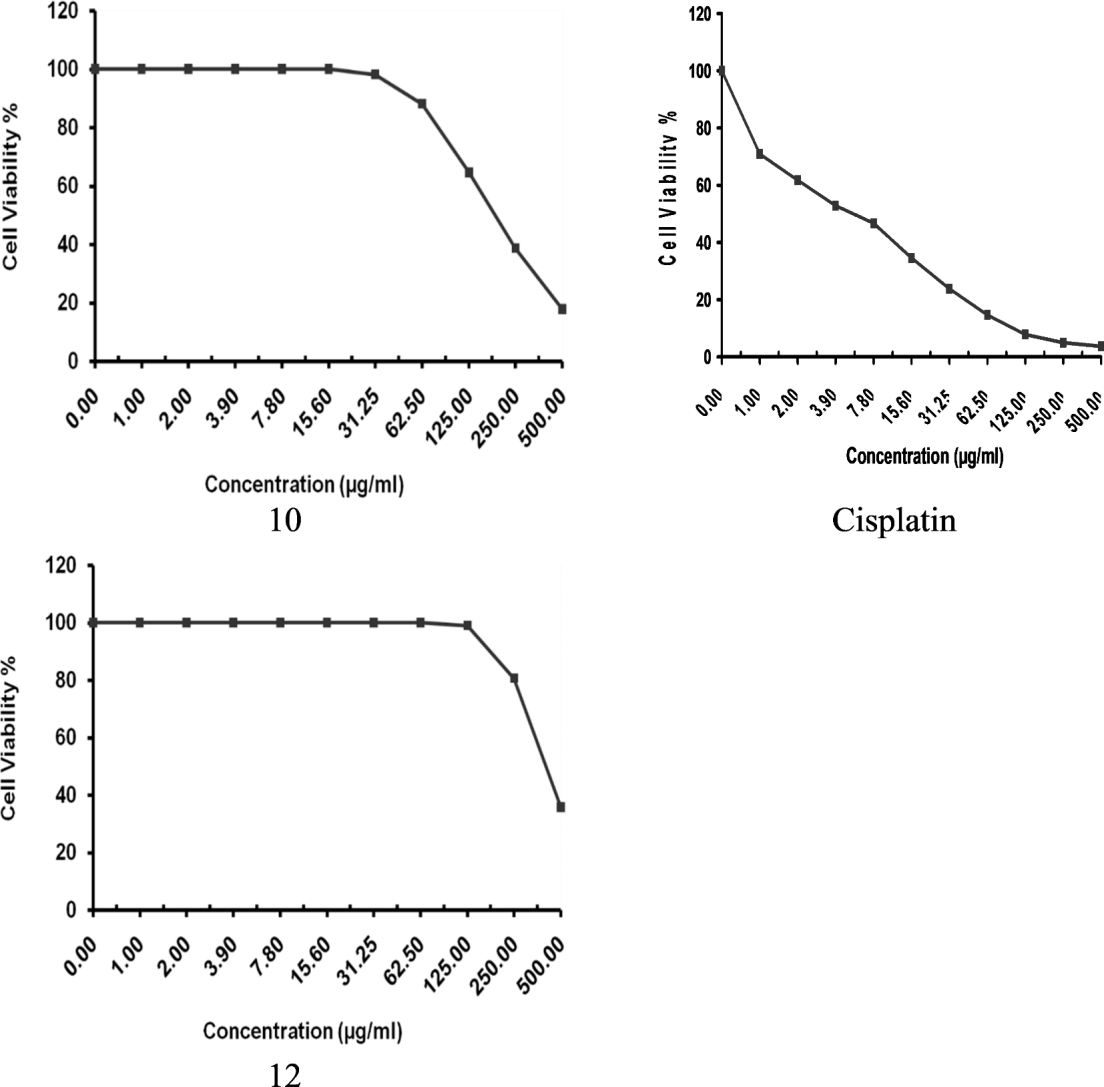
The antitumor activity of some synthesized pyrimidine compounds (**10** and **12**) against the breast cancer cell line MCF-7 as compared to cisplatin standard antitumor as detected using MTT assay.

One of the objectives of this study was to analyze the effectiveness of two newly synthesized pyrimidine derivatives, compounds **10** and **12**, in inhibiting tumor growth in the MCF-7 breast carcinoma cell line. To evaluate their efficacy, the MTT assay was utilized, and the results were compared to those obtained with the standard antitumor drug cisplatin (Table 5 and Fig. 11). The findings indicated that compound **12** exhibited a minimal level of antitumor activity, as evidenced by its IC50 value of 420.75 µg/ml, suggesting relatively lower efficacy in inhibiting tumor growth. In contrast, compound 10 demonstrated moderate antitumor activity against the MCF-7 cell line, with an IC50 value of 195.40 µg/ml. Notably, when compared to cisplatin, which displayed an IC50 value of 5.69 µg/ml, compound **10** exhibited relatively moderate antitumor activity, thereby making it a promising candidate for further in vitro investigations to ascertain its potency in inhibiting tumor growth in other cell lines.

**Table 5:** The antitumor activity of some synthesized pyrimidine compounds **10** and** 12** against the breast cancer cell line MCF-7 as compared to cisplatin standard antitumor as detected using MTT assay.

Conc. (µg/ml)	Compounds			
	10		12	
	Viability (%)	Inhibition (%)	Viability (%)	Inhibition (%)
500.0	17.85 ± 1.39	82.15	35.79 ± 2.37	64.21
250.0	38.71 ± 2.05	61.29	80.62 ± 1.46	19.38
125.0	64.56 ± 1.42	35.44	98.96 ± 0.42	1.04
62.5	88.07 ± 0.69	11.93	100	0
31.25	98.12 ± 0.44	1.88	100	0
15.6	100	0	100	0
7.8	100	0	100	0
3.9	100	0	100	0
2.0	100	0	100	0
1.0	100	0	100	0
0.0	100	0	100	0
IC50 (µg/ml)	195.40 ± 6.93		420.75 ± 17.28

Pyrimidine compounds have been reported as promising antitumor compounds, making them suitable anticancer candidates. Through various mechanisms, these compounds affect tumor development and progression processes. Pyrimidine derivatives block the DNA-synthesis enzyme thymidylate synthase (TS)^19^. These compounds impede TS, preventing DNA replication by blocking thymidine synthesis. This disruption damages DNA and inhibits cell division, preventing tumor growth. Pyrimidine derivatives also inhibit EGFR and VEGFR tyrosine kinases^20,21^. Tumor cell proliferation, angiogenesis, and metastasis depend on these kinases. Pyrimidine derivatives block these signaling pathways, preventing cancer cell proliferation and blood vessel development^22^. Additionally, several pyrimidine derivatives were reported to induce cancer cell death by activating programmed cell death, as they trigger apoptotic pathways that eradicate cancer cells^23^. These properties highlight their potential as targeted therapies against various types of cancers, although further research and clinical trials are necessary to fully explore their efficacy and optimize their therapeutic potential.

### Structure activity relationship (SAR) of the synthesized compounds

The outcomes of biological assays conducted on the synthesized derivatives of 4,6-dimethylpyrimidine-2-thiol hydrochloride offer valuable insights into the Structure–Activity Relationship (SAR) of these compounds. Specifically, Compound 1, featuring a thiol group on the pyrimidine ring, exerts a substantial influence on the compound's antimicrobial efficacy. Thiol groups are recognized for their adeptness in engaging with a spectrum of biological entities, encompassing proteins and enzymes, thereby impacting microbial proliferation and viability^40^. Moreover, the hydrophobic characteristics of the thiol group play a pivotal role in modulating the compound's capacity to infiltrate microbial membranes, thereby facilitating its ingress into microbial cells. These thiol groups can establish covalent bonds with pivotal enzymes crucial for microbial sustenance, thereby impeding their functionality and disrupting the microbial redox equilibrium, consequently bolstering the antimicrobial properties of the compound. The SAR observations concerning compounds 6 and 10 underscore how the phenyl ring can augment the antifungal and antibacterial efficacy of the synthesized pyrimidine derivatives^4^. Similarly, the SAR analysis of compound 12 delineates how the introduction of an ethyl group can enhance the antifungal and antibacterial effectiveness of the synthesized pyrimidine derivatives. Conversely, the restricted antimicrobial performance of compound 14 elucidates that the inclusion of p-tolyl may compromise the efficacy of the pyrimidine derivatives against the targeted pathogens.

Furthermore, owing to the radical scavenging attributes of the synthesized compounds, it is evident that compound 12 (ethyl substitution) and compound 6 (phenyl substitution) exhibit heightened scavenging activity compared to the start compound. The incorporation of an ethyl group into the pyrimidine ring is noted to enhance radical scavenging proficiency due to the electron-donating capabilities of ethyl groups, which potentially enhance the compound's ability to counteract radicals. Similarly, the addition of a phenyl group elevates radical scavenging efficacy owing to the aromatic nature of the phenyl group, which bolsters the compound's stability and reactivity towards radicals. Additionally, phenyl groups can facilitate electron delocalization, thereby influencing the radical scavenging capabilities of the compound.

Regarding anti-tumor effectiveness, compound 10 has demonstrated notable potency against the MCF-7 cell line, demonstrating moderate anti-tumor activity relative to cisplatin. The SAR analysis of pyrimidine sulfonothioate phenyl underscores the significance of substituting a phenyl group onto a pyrimidine scaffold, coupled with a sulfonothioate moiety. While the sulfonothioate group endows reactivity and specificity, the phenyl group contributes to lipophilicity and target affinity^41,42^. By comprehending the SAR intricacies of the modified pyrimidine derivatives, researchers can optimize their designs to elicit desired biological effects, spanning antimicrobial, antioxidant, and anticancer activities, through precise structural modifications and property tunings.

## Conclusion

In conclusion, the successful synthesis of pyrimidine benzothioate derivatives (2–5), pyrimidine sulfonylmethanone derivatives (6–9), and sulfonyl sulfonylpyrimidines (13–15) through benzoylation, sulfoxidation, and condensation processes marks a significant achievement. Characterized accurately using diverse analytical methods, these compounds demonstrated compelling biological activities. Compounds 1 and 6 exhibited notable antimicrobial efficacies, while compounds 12 and 6 displayed robust antioxidant properties. Additionally, compounds 10 and 12 revealed promising anti-tumor effects, with compound 12 exhibiting superior efficacy against the MCF-7 breast cancer cell line. These findings underscore the impact of chemical modifications on biological activity, offering valuable insights into further research and drug discovery endeavors.

## Data Availability

Data is provided within the manuscript or supplementary information files.
